# Efficacy and safety of canagliflozin in Japanese patients with type 2 diabetes: a randomized, double-blind, placebo-controlled, 12-week study†

**DOI:** 10.1111/dom.12149

**Published:** 2013-07-14

**Authors:** N Inagaki, K Kondo, T Yoshinari, N Maruyama, Y Susuta, H Kuki

**Affiliations:** 1 Department of Diabetes and Clinical Nutrition, Kyoto University Graduate School of MedicineKyoto, Japan; 2 Mitsubishi Tanabe Pharma CorporationTokyo, Japan

**Keywords:** SGLT2 inhibitor

## Abstract

**Aims** We examined the efficacy, safety and tolerability of canagliflozin, a sodium glucose co-transporter 2 inhibitor, in Japanese patients with type 2 diabetes (T2DM) undergoing diet and exercise therapy.

**Methods** Patients aged 20–80 years with T2DM diagnosed ≥3 months previously, and HbA1c of 6.9–9.9% were randomized to 50, 100, 200 or 300 mg canagliflozin or placebo once daily for 12 weeks. The primary and secondary endpoints were changes in HbA1c, fasting plasma glucose (FPG), urinary glucose/creatinine and postprandial glycaemic parameters following a meal test. The safety assessments included adverse events (AEs) and clinical laboratory tests.

**Results** Overall, 383 patients were randomized to receive either placebo (n = 75), or 50 mg (n = 82), 100 mg (n = 74), 200 mg (n = 77) or 300 mg canagliflozin (n = 75). At week 12, significant reductions in HbA1c were observed in all canagliflozin groups relative to placebo (−0.61, –0.80, –0.79 and −0.88% for 50, 100, 200 and 300 mg, respectively, versus +0.11% for placebo; all, p < 0.01). FPG and postprandial glycaemic parameters improved significantly in the canagliflozin groups. Body weight was significantly decreased by canagliflozin. No deaths or drug-related serious AEs were reported. There was no dose-dependent increase in the incidence of AEs in the canagliflozin groups. The incidence of hypoglycaemia was low; episodes were not severe or dose dependent. Canagliflozin did not affect serum creatinine levels or the urinary albumin/creatinine ratio.

**Conclusions** Treatment with canagliflozin for 12 weeks significantly improved glycaemic control and reduced body weight in Japanese patients with T2DM. Canagliflozin was well tolerated.

## Introduction

There are an estimated 366 million patients with diabetes worldwide, including 10.7 million in Japan 1. The prevalence of type 2 diabetes worldwide is expected to increase significantly over the next 20 years 1. Current treatment recommendations focus on improving diet and exercise, followed by monotherapy with an antihyperglycaemic drug 2–3.

Numerous studies, including the ADOPT trial 4, have shown that β-cell function continues to decline and that type 2 diabetes progressively worsens over time. Many existing treatments exert their effects by stimulating insulin secretion or by improving insulin action. However, such effects may be limited in patients with progressively deteriorating β-cell function. Other limitations of current therapies include hypoglycaemia, weight gain, peripheral oedema and gastrointestinal side effects, while many patients wish to avoid the side effects and inconvenience of injectable agents. Therefore, new therapeutic targets are needed to overcome or avoid the limitations associated with current drugs.

In healthy humans, virtually all of the filtered glucose is re-absorbed at plasma glucose (PG) levels of up to ∼10 mmol/l—the renal threshold for glucose (RT_G_)—at which point glucose transport reaches saturation 5. Above the RT_G_, the urinary glucose concentration increases proportionally to PG 6.

Sodium glucose co-transporter 2 (SGLT2) is a glucose transporter expressed in the proximal renal tubules, and is responsible for the majority of glucose re-absorption from urine. Its activity is also independent of insulin 7. Enhanced expression of SGLT2 and increased glucose uptake were reported in animal models of diabetes and in patients with diabetes, suggesting that the kidney plays important roles in the maintenance and progression of hyperglycaemia 8. Accordingly, inhibitors of SGLT2 were developed to lower the RT_G_ and enhance urinary glucose excretion (UGE) 7–11.

Canagliflozin (TA-7284 and JNJ-28431754; Mitsubishi Tanabe Pharma Corporation/Janssen Research & Development, LLC) is a potent, selective inhibitor of SGLT2 12.

One phase 1 study in healthy men showed that a single dose of canagliflozin (in the morning) of up to 800 mg per day significantly and dose-dependently increased 24-h UGE and dose-dependently reduced RT_G_ 13. The incidence of adverse events (AEs) was low, with the majority of AEs being mild in severity.

In patients with type 2 diabetes, treatment with canagliflozin (100 mg once daily or 300 mg twice daily) for 28 days as an add-on to insulin reduced RT_G_, increased UGE, and reduced HbA1c, fasting plasma glucose (FPG) and body weight compared with placebo 14. In a subsequent 12-week study, canagliflozin as an add-on to metformin significantly improved glycaemic control, with a low incidence of hypoglycaemia and with significant weight loss compared with placebo in patients with type 2 diabetes 15. In another study, treatment with 100 or 300 mg canagliflozin once daily for 26 weeks in patients with type 2 diabetes who were on diet and exercise therapy alone significantly reduced HbA1c, FPG, 2-h postprandial PG, body weight and systolic blood pressure compared with placebo 16.

However, these three studies were conducted mainly in obese Caucasian patients. Therefore, studies are needed to assess the efficacy and safety profiles of canagliflozin in other ethnic groups. The aim of this study was to determine the efficacy, safety and optimal doses of canagliflozin for the treatment of type 2 diabetes in Japanese patients.

## Methods

### Patients

Patients aged 20–80 years who were diagnosed with type 2 diabetes at least 3 months before the run-in period and who had HbA1c levels of 6.9–9.9% at the start of the run-in period were eligible for this study. Patients were to have undergone diet and exercise therapy, with no change in their regimen for ≥8 weeks before the study. Patients previously treated with antihyperglycaemic drugs were also eligible if their treatment was discontinued during a washout period after they had provided informed consent. Exclusion criteria included history of or current serious diabetic complications (e.g. proliferative diabetic retinopathy, stage 3 or later overt nephropathy, diabetic ketoacidosis or serious diabetic neuropathy), FPG ≥270 mg/dl (1 mg/dl FPG = 0.0555 mmol/l), indication for insulin therapy, hereditary glucose-galactose malabsorption or renal glycosuria. Additional inclusion/exclusion criteria are listed in the Supporting Information. The study was conducted in accordance with the ethical principles of the Declaration of Helsinki, the Pharmaceutical Affairs Law of Japan, Good Clinical Practice (GCP), and the approved study protocol. The study was approved by institutional review boards at each participating site. All patients provided written informed consent before entering the washout or the run-in period.

### Study Design, Treatments and Blinding

This was a multicentre, randomized, placebo-controlled, parallel-group study. After giving informed consent, patients treated with diet and exercise entered a single-blind 4-week placebo run-in period (with visits at weeks 2 and 4). Patients who were previously treated with one or more antihyperglycaemic drugs discontinued these agents after providing informed consent and entered a washout period of ≥8 weeks before starting the single-blind run-in period. Eligibility criteria were checked at the start and end of the run-in period. At the end of this period, eligible patients were randomized using a block allocation method into one of five groups (ratio 1 : 1 : 1 : 1 : 1) to receive placebo or one of four doses of canagliflozin (50, 100, 200 or 300 mg once daily) for 12 weeks. Randomization was conducted by a central committee, which provided the investigators at each site with randomization codes stored in sealed envelopes. The randomization code was not to be broken until data entry had been completed or unless needed in an emergency. Investigators and patients were blinded to the study drug received during the treatment phase. Visits were scheduled at weeks 4, 8 and 12 in the treatment phase, with a follow-up visit 2 weeks after the treatment phase. The patients did not receive the study drug during the follow-up phase. In terms of concomitant treatments, antihyperglycaemic drugs were prohibited after randomization until the end of the follow-up period. Diet and exercise interventions were to be continued without modification after randomization until the end of the follow-up period. This trial was registered at http://ClinicalTrials.gov (NCT01022112).

### Efficacy Outcomes

The primary endpoint was the change in HbA1c from the last day of the run-in period (baseline) to the end of the treatment period. Secondary endpoints included change in FPG, percentages of patients with HbA1c <7.0%, changes in urinary glucose/creatinine ratio, body weight, body mass index (BMI), waist circumference, lipid levels, blood pressure, insulin and proinsulin levels, homeostasis model assessment of β-cell function (HOMA-β) and meal tolerance-related parameters.

### Meal Tolerance Test

At baseline and week 12, patients underwent a meal tolerance test after a ≥10-h fast (water and calorie-free drinks were permitted). After basal blood sampling and completely emptying their bladder, the patients consumed (within 10 min) a standard test meal (approximately 500 kcal; 60% carbohydrate, 25% fat and 15% protein). Blood samples were obtained at 30, 60, 90 and 120 min after starting the meal, and urine samples were also collected. At 120 min, the patients were asked to completely empty their bladder.

### Treatment Compliance

To examine treatment compliance, the number of tablets remaining at each visit was counted, and the investigator verbally discussed treatment adherence with the patient.

### Safety

AEs and safety assessments, including vital signs, 12-lead electrocardiography, clinical laboratory tests (blood chemistry, haematology, coagulation, bone markers and urinalysis), and hypoglycaemic symptoms, were recorded throughout the study. AEs were classified according to system organ class and preferred term (MedDRA/J version 13.0) and were evaluated in terms of their potential relationship with the study drug (no causal relationship or possible causal relationship) and severity (mild, moderate or severe).

### Statistical Methods

The sample size calculation is described in the Supporting Information online. Primary and secondary analyses were conducted in the full analysis set (FAS), defined as all allocated patients, excluding patients who did not receive any study drug or who did not have any efficacy data after entering the treatment phase. In the event of missing data for efficacy variables, the last observation carried forward (LOCF) approach was used to impute missing values in the FAS analyses. Safety parameters were evaluated in the safety analysis set, defined as all patients, except those who did not receive any study drug during the treatment phase or who did not have any safety data during the treatment phase. The primary and secondary endpoints were examined descriptively and by analysis of covariance (ancova) with treatment group as a fixed factor and value at baseline as a covariate. Comparisons between canagliflozin and placebo at week 12 (with LOCF) and significance tests were performed based on the differences between the least squares (LS) means (the adjusted mean change from baseline obtained from ancova) for each treatment. For categorical variables, the percentage of patients was determined with 95% CIs (confidence intervals). The incidences of AEs and adverse drug reactions (ADRs) were determined descriptively as the number and proportion of patients. According to the closed testing procedures, the analysis of multiple comparisons of the primary endpoint was performed with a two-sided significance level of 0.05. Because the significance level was not controlled in multiple comparisons of data other than the primary endpoint, the p values for these comparisons are nominal. For baseline imbalances in patient characteristics, the significance level was set at 0.15 (two-sided).

## Results

### Patients

**Figure 1:**
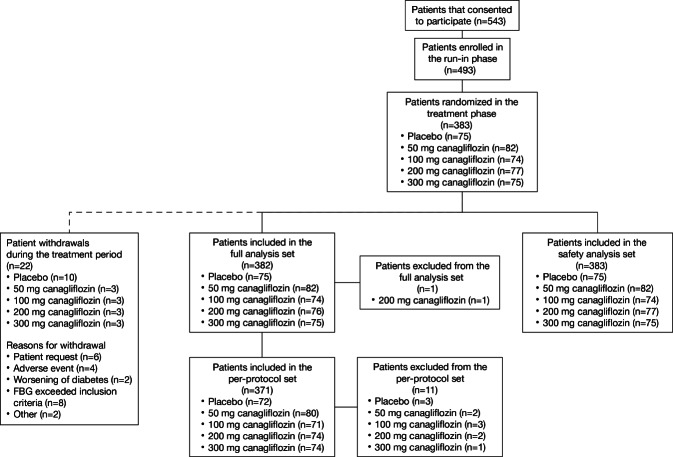
Patient disposition.

The disposition of patients is summarized in Figure 1. Five-hundred and forty-three patients consented to participate, 493 entered the run-in period and 383 were randomized and treated with either placebo (n = 75), or 50 mg (n = 82), 100 mg (n = 74), 200 mg (n = 77) or 300 mg canagliflozin (n = 75). Overall, 160 patients withdrew from the study before randomization for the following reasons: patient’s request (n = 23), did not meet eligibility criteria (n = 97), FPG decreased below the specified range (n = 10), worsening of diabetes (n = 2), experienced an AE (n = 1) or other reasons (n = 27). Twenty-two patients withdrew after randomization because the FPG was beyond the level specified in the exclusion criteria (n = 8), at the patient’s request (n = 6), because of an AE (n = 4), worsening of diabetes (n = 2) or other reasons (n = 2). The full analysis and safety analysis sets consisted of 382 and 383 patients, respectively.

Patient characteristics were generally well balanced among the five groups, with no marked differences among treatment groups (Table1) except for height, body weight and presence/absence of neuropathy or hypertension, which were unbalanced among the five groups at p < 0.15. Ancova was performed using baseline HbA1c and these imbalanced factors as covariates and showed that the changes in HbA1c were unaffected by adjustment for any of these variables. Of the 382 patients included in the full analysis set, 260 (68.1%) were male, and their mean ± standard deviation age was 57.4 ± 10.6 years, BMI was 25.70 ± 4.23 kg/m^2^ and HbA1c was 8.09 ± 0.82%. Just under half of the patients (44.0%) had previously received antidiabetic drugs, including sulfonylureas (16.8%) and biguanides (14.9%); these patients entered a washout period of ≥8 weeks after providing informed consent and before starting the run-in period.

**Table 1:** Patient characteristics (full analysis set)

		Canagliflozin					
Variable	Placebo	50 mg	100 mg	200 mg	300 mg	All patients	p-value
N	75	82	74	76	75	382	
Males, n (%)	54 (72.0)	50 (61.0)	52 (70.3)	49 (64.5)	55 (73.3)	260 (68.1)	0.4054*
Age, years	57.7 ± 11.0	57.4 ± 10.8	57.7 ± 10.5	57.0 ± 10.7	57.1 ± 10.1	57.4 ± 10.6	0.9883†
Height, cm	165.30 ± 9.31	161.44 ± 9.18	163.38 ± 8.73	164.04 ± 8.94	165.81 ± 9.19	163.95 ± 9.16	0.0238†
Weight, kg	72.56 ± 15.36	65.77 ± 13.56	68.61 ± 14.86	68.97 ± 14.50	71.30 ± 12.19	69.38 ± 14.25	0.0303†
BMI, kg/m^2^	26.41 ± 4.34	25.11 ± 4.13	25.61 ± 4.64	25.51 ± 4.30	25.89 ± 3.68	25.70 ± 4.23	0.4042†
Waist circumference, cm	92.0 ± 10.9	88.0 ± 9.7	89.8 ± 11.7	89.9 ± 9.8	90.0 ± 9.7	89.9 ± 10.4	0.2071†
FPG, mg/dl‡	170.7 ± 31.9	161.4 ± 34.6	161.0 ± 32.1	165.9 ± 31.4	169.1 ± 34.2	165.6 ± 32.9	0.2426†
HbA1c, %	7.99 ± 0.77	8.13 ± 0.78	8.05 ± 0.86	8.11 ± 0.88	8.17 ± 0.81	8.09 ± 0.82	0.7309†
eGFR, ml/min/1.73 m^2^	83.0 ± 16.5	83.5 ± 16.1	86.9 ± 15.5	83.8 ± 15.0	86.9 ± 15.2	84.8 ± 15.7	0.3404†
Diabetic complications, n (%)							
Any	10 (13.3)	14 (17.1)	9 (12.2)	6 (7.9)	7 (9.3)	46 (12.0)	0.4292*
Retinopathy	3 (4.0)	4 (4.9)	3 (4.1)	1 (1.3)	4 (5.3)	15 (3.9)	0.7417*
Neuropathy	0 (0.0)	4 (4.9)	1 (1.4)	1 (1.3)	0 (0.0)	6 (1.6)	0.0828*
Nephropathy	7 (9.3)	9 (11.0)	5 (6.8)	4 (5.3)	4 (5.3)	29 (7.6)	0.5800*
Non-diabetic complications, n (%)							
Hypertension	37 (49.3)	34 (41.5)	39 (52.7)	33 (43.4)	25 (33.3)	168 (44.0)	0.1452*
Dyslipidaemia	41 (54.7)	52 (63.4)	44 (59.5)	49 (64.5)	52 (69.3)	238 (62.3)	0.4210*
Previous antidiabetic drugs, n (%)							
Any	25 (33.3)	38 (46.3)	35 (47.3)	31 (40.8)	39 (52.0)	168 (44.0)	0.1820*
Sulfonylureas	11 (14.7)	14 (17.1)	13 (17.6)	8 (10.5)	18 (24.0)	64 (16.8)	n/d
Thiazolidinediones	6 (8.0)	10 (12.2)	3 (4.1)	2 (2.6)	7 (9.3)	28 (7.3)	n/d
Rapid-acting insulin secretagogues	1 (1.3)	4 (4.9)	6 (8.1)	7 (9.2)	3 (4.0)	21 (5.5)	n/d
α-Glucosidase inhibitors	7 (9.3)	5 (6.1)	5 (6.8)	6 (7.9)	9 (12.0)	32 (8.4)	n/d
Biguanides	7 (9.3)	14 (17.1)	14 (18.9)	14 (18.4)	8 (10.7)	57 (14.9)	n/d

Values are means ± standard deviation or n (%). BMI, body mass index; FPG, fasting plasma glucose; eGFR, estimated glomerular filtration rate; n/d, no data.

* *χ*^2^ test.

† Analysis of variance.

‡ FPG conversion factor: 1 mg/dl = 0.0555 mmol/l.

Treatment compliance during the randomized treatment phase ranged from 97.4 to 99.1% among the five groups.

### Glycaemic Control

**Figure 2:**
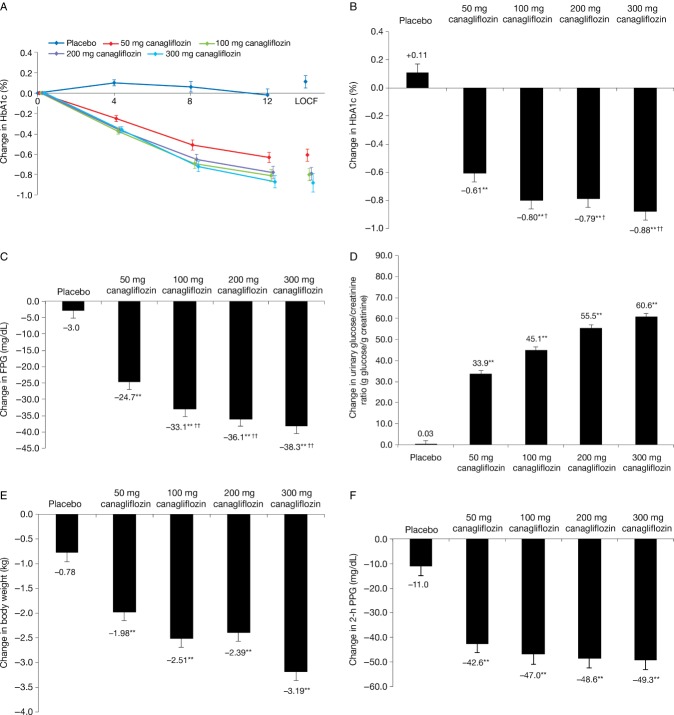
Effects of canagliflozin on the time course of changes (A) and changes from the start of the treatment phase to week 12 (B) in HbA1c. Changes in fasting plasma glucose (FPG; C), urinary glucose/creatinine ratio (D), body weight (E) and 2-h postmeal plasma glucose (PPG; F) from the start of the treatment phase to week 12. Values are LS means ± standard error. For all analyses, the last observation was carried forward. **p < 0.01 versus placebo; ^†^p < 0.05 and ^††^p < 0.01 versus 50 mg canagliflozin. The time courses of HbA1c, FPG, urinary glucose/creatinine ratio and body weight are shown in figure S1. Glucose levels during the meal tolerance tests at baseline and at week 12 are also shown in figure S2.

Figure 2A, B shows the changes in HbA1c over time and the change from baseline to week 12 (with LOCF) in each of the five groups. HbA1c levels measured at each time are shown in figure S1A. Improvements in HbA1c were observed as early as week 4 in the canagliflozin groups. The mean changes in HbA1c in the 50, 100, 200 and 300 mg canagliflozin groups (−0.61, –0.80, –0.79 and −0.88%, respectively) differed significantly from that in the placebo group (+0.11%; all, p < 0.01). The changes in HbA1c from baseline to week 12 (with LOCF) were significantly greater in the 100, 200 and 300 mg groups than in the 50 mg group (all, p < 0.05). More patients in the 50, 100, 200 and 300 mg canagliflozin groups achieved HbA1c < 7% at week 12/LOCF compared with the placebo group; specifically, HbA1c < 7% at week 12/LOCF was achieved by 17 (21.0%), 24 (33.8%), 21 (29.2%), 30 (40.5%) and 4 (5.7%) patients in these groups, respectively.

The reductions in FPG (figure 2C) from baseline to week 12 (with LOCF) were significantly greater in all four canagliflozin-treated groups compared with placebo (all, p < 0.01). The reductions in FPG (all, p < 0.01) were also significantly greater in the 100, 200 and 300 mg canagliflozin groups compared with the 50 mg group. FPG levels measured at each time are shown in figure S1B.

### Other Efficacy Variables

Figure 2D and figure S1C show the effects of canagliflozin on urinary glucose/urinary creatinine ratio (i.e. UGE) from baseline to week 12. All four doses of canagliflozin elicited significant increases in UGE compared with placebo (all, p < 0.01), and these increases were maximal at the first on-treatment visit (week 4), with UGE reaching a plateau thereafter. The increases in UGE also showed a dose-dependent trend, with the greatest increases observed in the 300 mg canagliflozin group.

As indicated in figures 2E and S1D, treatment with canagliflozin for 12 weeks (with LOCF) resulted in significantly greater reductions in body weight compared with placebo. These reductions in body weight were also dose dependent and were accompanied by small, but significant, reductions in waist circumference (Table2).

**Table 2:** Changes in waist circumference, lipids, blood pressure, insulin, proinsulin, proinsulin/insulin ratio, HOMA-β and meal tolerance tests from the start of the treatment phase to week 12 (with last observation carried forward)

		Canagliflozin			
Variable	Placebo	50 mg	100 mg	200 mg	300 mg
Waist circumference, cm	−0.59 ± 0.37	−1.59 ± 0.35*	−1.81 ± 0.37*	−1.83 ± 0.36*	−2.21 ± 0.37**
Insulin, μIU/ml†	−0.61 ± 0.45	−1.62 ± 0.43	−1.62 ± 0.45	−2.39 ± 0.44**	−2.88 ± 0.44**
Proinsulin, pmol/l	0.41 ± 0.82	−5.19 ± 0.79**	−6.65 ± 0.83**	−7.16 ± 0.82**	−7.07 ± 0.82**
Proinsulin/insulin ratio	0.0214 ± 0.0185	−0.0482 ± 0.0177**	−0.0500 ± 0.0186**	−0.0326 ± 0.0185*	−0.0155 ± 0.0186
HOMA-β, %	−1.61 ± 1.92	2.97 ± 1.84	6.98 ± 1.94**	4.80 ± 1.92*	2.81 ± 1.92
HDL-C, mg/dl‡	0.3 ± 0.8	2.2 ± 0.8	4.6 ± 0.8**	5.5 ± 0.8**	5.0 ± 0.8**
LDL-C, mg/dl‡	−0.9 ± 2.2	4.6 ± 2.1	4.9 ± 2.2	8.1 ± 2.2**	5.5 ± 2.2*
TG, mg/dl§	−1.5 ± 6.4	−10.7 ± 6.1	−16.9 ± 6.4	−16.7 ± 6.3	−14.5 ± 6.3
TC, mg/dl‡	−2.3 ± 2.5	5.3 ± 2.3*	7.3 ± 2.5**	11.9 ± 2.4**	8.0 ± 2.5**
LDL-C/HDL-C ratio	−0.0074 ± 0.0407	−0.0009 ± 0.0390	−0.0640 ± 0.0410	−0.0594 ± 0.0405	−0.0725 ± 0.0408
SBP, mmHg	−1.2 ± 1.2	−5.8 ± 1.2**	−7.1 ± 1.2**	−9.3 ± 1.2**	−8.7 ± 1.2**
DBP, mmHg	−0.9 ± 0.9	−2.2 ± 0.8	−3.9 ± 0.9*	−5.1 ± 0.8**	−4.2 ± 0.8**
2-h postmeal insulin, μIU/ml¶	−1.66 ± 2.20	−12.56 ± 1.99**	−12.07 ± 2.13**	−15.54 ± 2.07**	−14.27 ± 2.10**
2-h postmeal UGE, g¶	−0.55 ± 0.57	8.16 ± 0.51**	8.25 ± 0.55**	8.16 ± 0.53**	9.54 ± 0.53**
Plasma glucose	−14.22 ± 6.02	−77.93 ± 5.42**	−96.20 ± 5.80**	−108.36 ± 5.64**	−110.49 ± 5.68**
AUC_0–2h_, mg·h/ml¶					
Plasma insulin	−4.23 ± 2.90	−18.53 ± 2.62**	−25.13 ± 2.78**	−32.43 ± 2.70**	−33.24 ± 2.76**
AUC_0–2h_, μIU·h/ml¶					

Values are LS means ± standard error. The p-values were calculated without adjusting for multiple comparisons between groups; HOMA-β, homeostasis model assessment of β-cell function; HDL-C, high-density lipoprotein–cholesterol; LDL-C, low-density lipoprotein–cholesterol; TG, triglyceride; TC, total cholesterol; SBP, systolic blood pressure; DBP, diastolic blood pressure; UGE, urinary glucose excretion; AUC, area under the curve.

† Insulin conversion factor: 1 μIU/ml = 6.945 pmol/l.

‡ HDL-C, LDL-C and TC conversion factor: 1 mg/dl = 0.0259 mmol/l.

§ TG conversion factor: 1 mg/dl TG = 0.0113 mmol/l.

¶ Measured after the meal tolerance test.

* p < 0.05 and

** p < 0.01 versus placebo.

Table2 also summarizes the effects of canagliflozin on other clinically relevant parameters, including change from baseline to week 12 (with LOCF) for high-density lipoprotein–cholesterol (HDL-C), triglyceride, insulin and proinsulin levels, proinsulin/insulin ratio, HOMA-β and blood pressure. Overall, all four doses of canagliflozin improved these parameters compared with placebo. Modest increases were observed in low-density lipoprotein–cholesterol (LDL-C) and total cholesterol levels in the canagliflozin-treated groups compared with the placebo group, but the LDL-C/HDL-C ratio was decreased in all canagliflozin-treated groups.

### Meal Tolerance Test

Meal tolerance tests were performed at baseline and week 12 (Table2). Glucose levels measured immediately before and at the specified times after consuming the meal are shown in figure S2. All four doses of canagliflozin provided significant reductions from baseline to week 12 (with LOCF) in 2-h PPG (figure 2F) and 2-h postmeal insulin levels, and in the areas under the glucose and insulin concentration curves for 0–2 h (AUC_0–2h_). By contrast, 2-h postmeal UGE increased in the canagliflozin-treated groups from baseline to week 12 (with LOCF).

### Safety

#### AEs

Overall, 266 AEs occurred in 169 patients, including 43 AEs in 26 (34.7%) patients in the placebo group, 52 AEs in 37 (45.1%) patients in the 50 mg group, 60 AEs in 34 (45.9%) patients in the 100 mg group, 61 AEs in 38 (49.4%) patients in the 200 mg group and 50 AEs in 34 (45.3%) patients in the 300 mg group. There were no deaths. AEs leading to study discontinuation occurred only in 0–2 patients across the groups (lung adenocarcinoma, pollakiuria, oral discomfort and pruritus) (Table3). Table4 summarizes the most common treatment-emergent AEs in each group. Most of the AEs were mild; only one serious AE occurred (lung adenocarcinoma in one patient in the 50 mg group). Five patients with treatment-emergent AEs withdrew from the study although, in one of these, the primary reason for withdrawal was disease progression. The AEs reported in >3% patients were nasopharyngitis, increased blood ketone bodies, hypoglycaemia unawareness, hypoglycaemia, gastritis, periodontitis, upper respiratory tract infections and malaise, but there was no dose-dependent trend. Two vulvovaginal infections were reported in the canagliflozin group (one each of vulvovaginal candida infection in the 100 and 300 mg groups). Volume-related AEs (dry mouth, dehydration, dizziness and palpitation) were reported in 0–3 patients in the canagliflozin groups and in 1 patient in the placebo group. Pollakiuria was reported in three patients in the canagliflozin groups and in none of the patients in the placebo group. No urinary tract infections were reported in any group.

**Table 3:** Summary of adverse events

		Canagliflozin			
	Placebo	50 mg	100 mg	200 mg	300 mg
Adverse events, n (%)	26 (34.7)	37 (45.1)	34 (45.9)	38 (49.4)	34 (45.3)
Serious adverse events, n (%)	0 (0.0)	1 (1.2)	0 (0.0)	0 (0.0)	0 (0.0)
Adverse events leading to study discontinuation, n (%)	0 (0.0)	2 (2.4)	2 (2.7)	0 (0.0)	1 (1.3)

**Table 4:** Incidence of adverse events (≥3% of patients in any group) classified using the MedDRA

		Canagliflozin					
	Placebo	50 mg	100 mg	200 mg	300 mg
Variable	n (%)	n (%)	n (%)	n (%)	n (%)
Adverse events in ≥3% of patients in any group					
Infections and infestations					
Nasopharyngitis	10 (13.3)	8 (9.8)	10 (13.5)	8 (10.4)	9 (12.0)
Investigations					
Increased blood ketone bodies	2 (2.7)	4 (4.9)	5 (6.8)	9 (11.7)	6 (8.0)
Metabolism and nutrition disorders					
Hypoglycaemia unawareness	0 (0.0)	1 (1.2)	1 (1.4)	3 (3.9)	2 (2.7)
Hypoglycaemia	0 (0.0)	3 (3.7)	2 (2.7)	2 (2.6)	1 (1.3)
Gastrointestinal disorders					
Gastritis	3 (4.0)	0 (0.0)	1 (1.4)	0 (0.0)	1 (1.3)
Periodontitis	0 (0.0)	3 (3.7)	0 (0.0)	1 (1.3)	0 (0.0)
Respiratory, thoracic and mediastinal disorders					
Upper respiratory tract inflammation	1 (1.3)	0 (0.0)	5 (6.8)	3 (3.9)	1 (1.3)
General disorders and administration site conditions					
Malaise	0 (0.0)	3 (3.7)	0 (0.0)	0 (0.0)	0 (0.0)

No clinically meaningful changes in electrocardiograms were observed. Although canagliflozin was associated with a decrease in blood pressure, no postural hypotension was reported. No clinically meaningful changes in serum electrolytes were observed in any of the groups. Small increases in haemoglobin, haematocrit and blood urea nitrogen were observed in the canagliflozin-treated groups. The change in blood total ketone body concentration peaked at week 4, showing a trend towards a dose-dependent increase. Thereafter, the mean changes in the 200- and 300-mg groups decreased, and the mean changes at week 12 of the treatment period were similar at all doses of canagliflozin. There were no remarkable changes in serum creatinine or the urinary albumin/creatinine ratio, suggesting that canagliflozin did not impair kidney function. No cardiovascular events occurred in this study.

There were small increases in urinary N-terminal cross-linked telopeptide of type I collagen levels and serum C-terminal cross-linked telopeptide of type I collagen levels together with slight decreases in bone-specific alkaline phosphatase and 1,25-(OH)_2_ vitamin D levels from baseline to week 12 in the canagliflozin groups. The clinical relevance of these small changes is unknown and there were no AEs suggestive of changes in bone-related markers.

#### Hypoglycaemia

As expected based on the mechanism of action, the incidence rates of hypoglycaemic symptoms and hypoglycaemia reported as an AE (including hypoglycaemia unawareness) were low in all of the canagliflozin-treated groups (Table4); no patient in the placebo group experienced hypoglycaemia. There were no major differences in the incidence of these symptoms/events among the four canagliflozin-treated groups (Table4). All episodes of hypoglycaemia were mild.

## Discussion

In this study, treatment with canagliflozin for 12 weeks significantly improved glycaemic control in terms of HbA1c, FPG and 2-h PPG, as well as other clinically relevant parameters (e.g. body weight, blood pressure and lipid levels) in patients with type 2 diabetes undergoing diet and exercise therapy. The changes, particularly glycaemic control, were dose dependent, with greater improvements with higher doses (≥100 mg) of canagliflozin compared with 50 mg canagliflozin.

AEs occurred in less than half of the patients, and were mostly mild in severity, with only one serious AE that was judged unrelated to the study drug. Furthermore, the incidence of hypoglycaemia and vulvovaginal infections was low, with no evidence for a dose-dependent effect on AEs.

Several other clinical studies of canagliflozin 14,15 have been published recently. In the first of these 14, a 28-day study involving 29 patients with type 2 diabetes, 100 mg canagliflozin once daily and 300 mg canagliflozin twice daily significantly reduced RT_G_ and increased UGE, HbA1c, FPG and body weight compared with placebo. The second study 15, a 12-week study, showed that doses of 50, 100, 200 or 300 mg canagliflozin once daily, or 300 mg twice daily, achieved significant changes in HbA1c of −0.79, −0.76, −0.70, −0.92 and −0.95%, respectively, compared with −0.22% for placebo (all p < 0.001) and −0.74% for 100 mg sitagliptin once daily. The changes in HbA1c, FPG and body weight observed in that study were similar to those observed in this study, and were associated with significant increases in the urinary glucose/creatinine ratio. In those studies, patients either continued insulin therapy 14 or metformin 15. Canagliflozin as monotherapy was tested in the study by Stenlöf et al. 16, who reported that HbA1c decreased significantly by −0.77 and −1.03% in patients treated with 100 and 300 mg canagliflozin, respectively, compared with 0.14% in patients treated with placebo (both, p < 0.001). As observed in the study by Stenlöf et al. 16, a significant increase in HDL-C, a decrease in triglycerides and a small increase in LDL-C were observed in our study. The LDL-C/HDL-C ratio decreased slightly in both studies. The clinical relevance of these small changes in lipids needs to be assessed in a long-term study.

Dapagliflozin is another SGLT2 inhibitor currently in clinical development. Several studies of ≥12 weeks in duration have been reported 17,18. Patients in those studies received dapagliflozin or placebo alone 19; dapagliflozin, metformin or placebo alone 17; or dapagliflozin or placebo in combination with oral antihyperglycaemic drugs plus insulin 18. The results of those studies were generally similar to those for canagliflozin in this study.

The RT_G_ was reported to be increased in patients with type 2 diabetes 20, supporting the rationale for using SGLT2 inhibitors to treat this disease 10–11. Canagliflozin substantially reduced RT_G_ in both animal models 21 and humans 22, favouring glucose excretion rather than re-absorption. Indeed, in the clinical studies performed to date, canagliflozin significantly enhanced UGE adjusted for urinary creatinine levels.

This study revealed that canagliflozin improves β-cell function in terms of HOMA-β and proinsulin/insulin ratio. The study by Rosenstock et al. 15 also revealed that canagliflozin improves β-cell function, estimated by HOMA2-%B. In an animal study, SGLT2 deletion was found to preserve β-cell function by increasing β-cell mass in mice fed a high-fat diet for 4 weeks 23. Further studies are needed to examine the precise mechanisms and the role of glucose toxicity in influencing β-cell function in humans. Studies evaluating the long-term effects of canagliflozin on β-cell function, including after cessation of treatment, will be of particular interest.

## Limitations

Some limitations of this study warrant mention. First, the study was relatively short (12 weeks), which may limit the extent of HbA1c lowering. Second, the number of patients enrolled may have been too small to detect significant differences among the higher dose groups. Finally, this study was conducted in Japanese patients, limiting generalizability to other patient populations.

## Conclusions

The results of this study show favourable efficacy and safety profiles of canagliflozin monotherapy in Japanese patients with type 2 diabetes, particularly at doses ≥100 mg per day. Additional clinical studies of canagliflozin are warranted and are now underway to extend these findings, including the use of canagliflozin instead of or in combination with other antihyperglycaemic drugs. The results of such studies will help establish the indications for canagliflozin.

## Appendix

Study investigators—Dr Tetsuya Noto, Kyouritsu Hospital of Gastroenterological and Cardiovascular Medicine, Hakodate Kyouritsu Medical Corporation; Dr Masahiro Tsutsui, Social Medical Corporation, Takahashi Hospital; Dr Kazuko Takahashi, Takahashi Medical Clinic Medical Corporation; Dr Toshiya Okamoto, Okamoto Naika Clinic; Dr Makoto Sugiura, Touei Hospital, Tokoharu Specified Medical Corporation; Dr Tamayuki Koizumi, Ishikari Hospital, Pietakai Medical Corporation; Dr Tatsujiro Segawa, Ebetsu Clinic; Dr Kenichi Imamura, Imamura Clinic; Dr Mikihiko Kudo, Kudo Internal Medical Clinic; Dr Yoshihito Kaneko, Kaneko Naika Clinic; Dr Hiroshi Otani, Iwase General Hospital; Dr Kenji Yaginuma, Social Insurance Nihonmatsu Hospital; Dr Hajime Ishii, Kashinoki Medical Clinic Medical Corporation; Dr Akihiko Okano, Okano Clinic, Kishokai Medical Corporation; Dr Osamu Ogawa, Midorigaoka Ekimae Clinic, Jozenkai Medical Corporation; Dr Akiteru Takeuchi, Takeuchi Hospital, Shinkokai Medical Foundation; Dr Yoshiko Nakayama, Medical Corporation Asagaya Clinic; Dr Katsuhiko Yamada, Kosei Medical Clinic; Dr Kazuyuki Mizuyama, Dojin Memorial Medical Foundation, Meiwa Hospital; Dr Hideki Hanashi, Medical Corp. Seikoukai, New Medical Research System Clinic; Dr Kazuo Suzuki, Kenkokan Suzuki Clinic; Dr Mitsutoshi Kato, Kato Clinic of Internal Medicine; Dr Akira Numata, Ikebukuro Metropolitan Clinic; Dr Yasuyuki Enomoto, Nihonbashi Enomoto Internal Medicine; Dr Munechika Noguchi, Shinagawa East One Medical Clinic, Seitokukai Medical Corporation; Dr Shusaku Hosono, Hosono Clinic; Dr Keiko Tsunoda, Ekimae Tsunoda Clinic; Dr Hidehiko Honda, Honda Hidehiko Clinic; Dr Nobuki Wakao, Medical Corp. Seikoukai, Kasai Diabetes Clinic; Dr Kazuo Kanno, Kanno Naika; Dr Arihiro Kiyosue, Tokyo-Eki Center-Building Clinic; Dr Ikuro Matsuba, Matsuba Clinic; Dr Hyeteok Kim, Yokohama Minoru Clinic, Shintokai Medical Corporation; Dr Ryuji Suzuki, Kokan Clinic, Kokankai Medical Corporation; Dr Yoji Takatsuka, Motomachi Takatsuka Naika Clinic; Dr Taro Asakura, Tsuruma Kaneshiro Diabetes Clinic, Yuga Medical Corporation; Dr Emi Kose, Sato Hospital, Yukokai Medical Corporation; Dr Shingo Tsuji, Osaka SEMPOS Hospital, Japanese Seamen’s Insurance Foundation (SEMPOS); Dr Sadahiro Sempuku, Sempuku Clinic; Dr Hiroya Tanaka, Tanaka Naika; Dr Ken Yaga, Yamaguchi Rosai Hospital, Japan Labour Health and Welfare Organization; Dr Shinya Fujii, Fujii Clinic; Dr Masamitsu Inoue, Inoue Internal Medical Clinic, Kenseikai Medical Corporation; Dr Tsuyoshi Tao, Tao Internal Medicine Clinic Medical Corporation; Dr Naoko Aga, Yashima General Hospital; Dr Toshihiko Inoue, Sanuki Municipal Hospital; Dr Tetsuji Niiya, Matsuyama Shimin Hospital; Dr Kazuaki Nakai, Ehime Rosai Hospital, Japan Labour Health and Welfare Organization; Dr Toshihiko Hashimoto, Wellness Tenjin Clinic, Hakuaikai Medical Treatment Corporate Foundation; Dr Masamichi Gushiken, Gushiken Cardiovascular Medical Clinic; Dr Tomoichiro Kuda, Urasoe General Hospital Healthcare Center.

## Supporting Information

Appendix S1 Eligibility criteria and the method for sample size calculation are provided in the Supporting Information.

Figure S1 HbA1c (A), FPG (B), urinary glucose/creatinine ratio (C) and body weight (D) measured at the start (except for urinary glucose/creatinine ratio and body weight) and end of the run-in period and during the 12-week treatment period. HbA1c, haemoglobin A1c; NGSP, National Glycohemoglobin Standardization Program; FPG, fasting plasma glucose; UGE, urinary glucose excretion; CRE, creatinine.

Figure S2 Plasma glucose levels measured during the meal tolerance tests performed at baseline (end of the run-in period) and atweek 12 in the treatment period in the placebo (A), 50mg (B), 100 mg (C), 200 mg (D) and 300 mg (E) canagliflozin groups. The meal was consumed at 0 min.
