# Supplementary material for: Efficacy and safety of canagliflozin in Japanese patients with type 2 diabetes: a randomized, double-blind, placebo-controlled, 12-week study
Source: Diabetes Obes Metab. 2013 Jul 14;15(12):1136–45. doi: 10.1111/dom.12149 (PMC3906835; doi:10.1111/dom.12149)
Supplement: Appendix S1 — Eligibility criteria and the method for sample size calculation are provided in the Supporting Information. [file dom0015-1136-sd1.doc]

**Supporting Information**

**Appendix S1**

### Eligibility criteria

Patients with any of the following were excluded from the study: type 1 diabetes mellitus, diabetes caused by pancreatic injury, or secondary diabetes (e.g., associated with acromegaly or Cushing syndrome); concurrent/poorly controlled thyroid abnormality; estimated glomerular filtration rate <60 mL/min/1.73 m^2^ on the first day of the run-in period; concurrent anorexia or bulimia; recurrent urinary tract infection; triglyceride level ≥600 mg/dL on the first day of the run-in period; systolic blood pressure ≥160 mmHg and/or diastolic blood pressure ≥100 mmHg on the first or last day of the run-in period; myocardial infarction, unstable angina, or cerebrovascular disorder within 3 months before the start of the run-in period; previous or concurrent cardiac failure symptoms corresponding to New York Heart Association class III or IV; serious concurrent liver or kidney disease (e.g., requiring hospitalization for treatment or for which surgery is indicated); patients with alanine aminotransferase or aspartate aminotransferase levels ≥2.5 times the upper limit of normal on the first day of the run-in period; previous or concurrent malignant tumour (except patients without recurrence for ≥5 years, even with a past history of malignant tumour); previous or concurrent drug-related hypersensitivity such as shock or anaphylactoid symptoms; unwilling to use birth control during the study period; women who are pregnant, breastfeeding, or who may be potentially pregnant; participation in another clinical study or received treatment with an investigational product within 12 weeks of providing informed consent; prior treatment with canagliflozin; deemed unsuitable by the investigator (subinvestigator) for inclusion in the study for another reason.

### Sample size

The sample size was calculated based on values reported in a previously published study for differences in HbA1c versus placebo of −0.45%, −0.51%, −0.54%, and −0.71% for 50, 100, 200 and 300 mg canagliflozin, respectively, and a standard deviation of 0.85%. For analysis of covariance and the contrast test to compare between 100 mg canagliflozin and placebo with 90% power (two-sided), 67 patients were required in each group for a significance level of 5%. This sample size yielded a power of 81% for 50 mg canagliflozin versus placebo. Therefore, taking into account the potential for dropouts and discontinuations, 75 patients were to be enrolled in each group.
