# Supplementary figures and images for: Efficacy and safety of canagliflozin in Japanese patients with type 2 diabetes: a randomized, double-blind, placebo-controlled, 12-week study
Source: Diabetes Obes Metab. 2013 Jul 14;15(12):1136–45. doi: 10.1111/dom.12149 (PMC3906835; doi:10.1111/dom.12149)

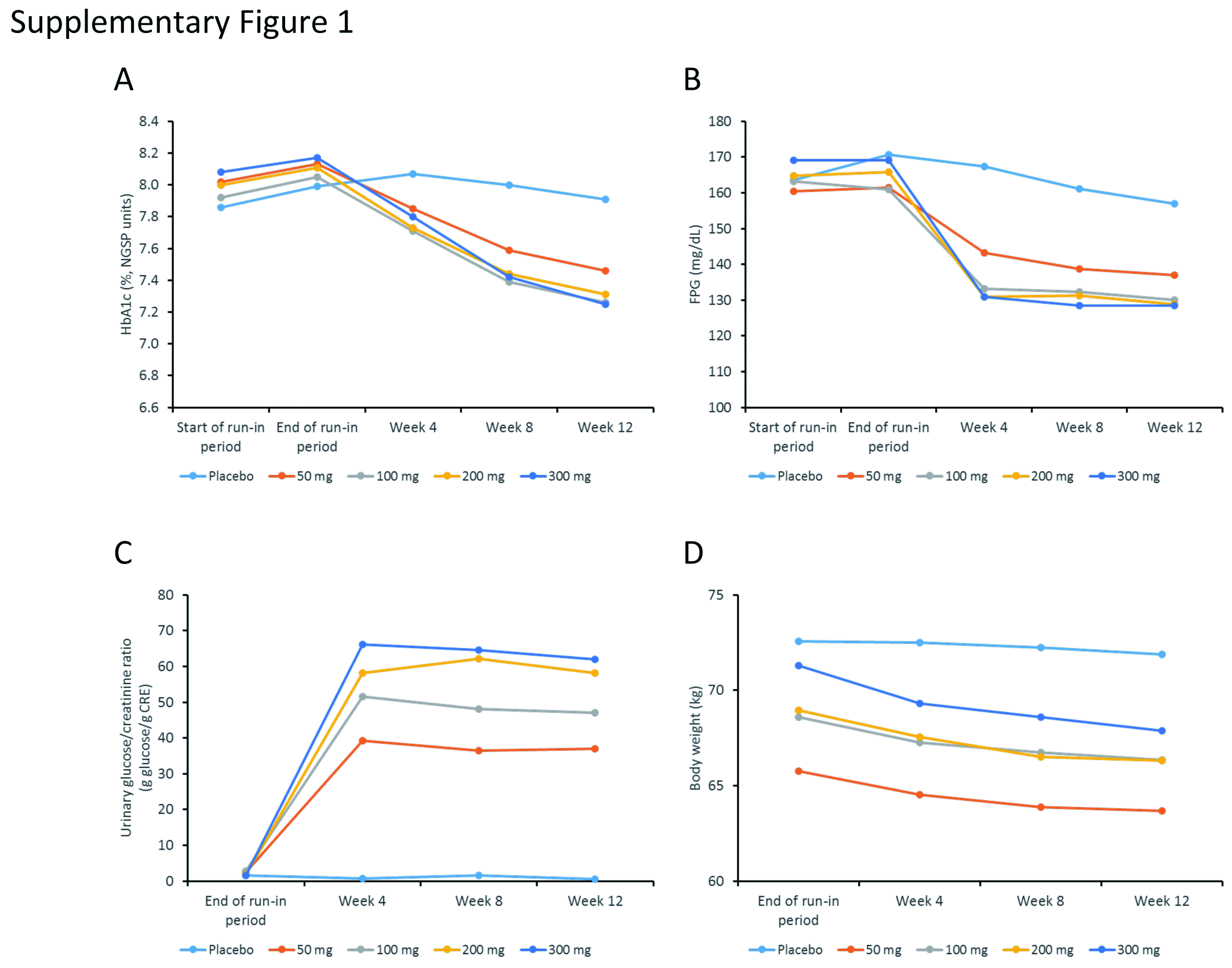

Supplement: Figure S1 — HbA1c (A), FPG (B), urinary glucose/creatinine ratio (C) and body weight (D) measured at the start (except for urinary glucose/creatinine ratio and body weight) and end of the run-in period and during the 12-week treatment period. HbA1c, haemoglobin A1c; NGSP, National Glycohemoglobin Standardization Program; FPG, fasting plasma glucose; UGE, urinary glucose excretion; CRE, creatinine. [file dom0015-1136-sd2.tif]

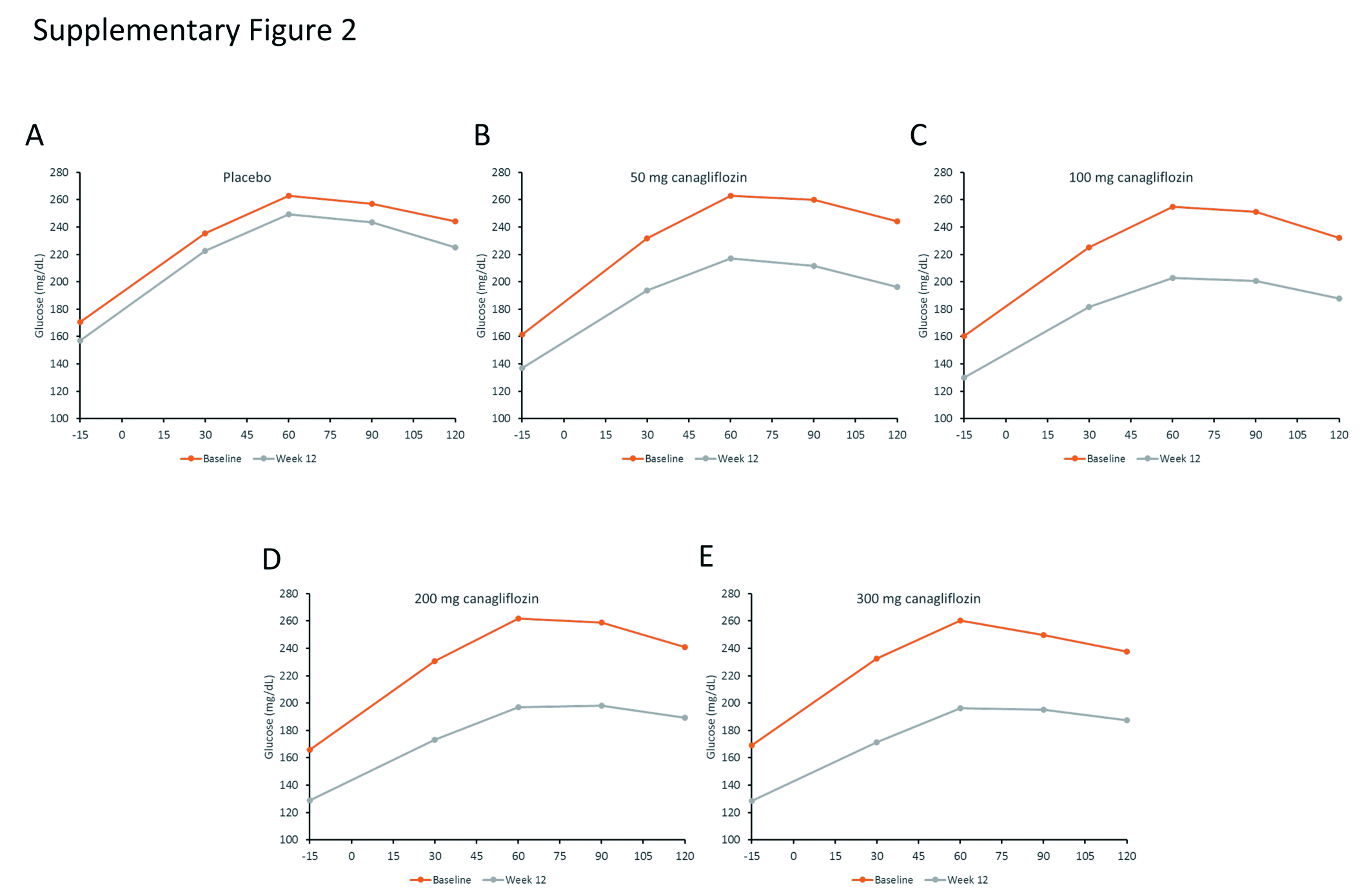

Supplement: Figure S2 — Plasma glucose levels measured during the meal tolerance tests performed at baseline (end of the run-in period) and atweek 12 in the treatment period in the placebo (A), 50mg (B), 100 mg (C), 200 mg (D) and 300 mg (E) canagliflozin groups. The meal was consumed at 0 min. [file dom0015-1136-sd3.tif]
